# 2-(2,4-Dichloro­phen­yl)-3-[5-(4-methoxy­phen­yl)-1,3,4-thia­diazol-2-yl]-1,3-thia­zolidin-4-one

**DOI:** 10.1107/S1600536808008465

**Published:** 2008-04-04

**Authors:** Rong Wan, Li-He Yin, Feng Han, Bin Wang, Jin-Tang Wang

**Affiliations:** a Department of Applied Chemistry, College of Science, Nanjing University of Technology, No. 5 Xinmofan Road, Nanjing 210009, People’s Republic of China

## Abstract

In the mol­ecule of the title compound, C_18_H_13_Cl_2_N_3_O_2_S_2_, the thia­zolidinone ring has an envelope conformation with the S atom displaced by 0.394 (3) Å from the plane of the other ring atoms. The thia­diazole ring is oriented at a dihedral angle of 7.40 (4)° with respect to the 4-methoxy­phenyl ring. Intra­molecular C—H⋯S, C—H⋯N and C—H⋯Cl hydrogen bonds result in the formation of two planar and two non-planar five-membered rings. The planar five-membered rings are oriented at a dihedral angle of 6.23 (3)°. The 2,4-dichloro­phenyl ring is oriented at dihedral angles of 84.21 (4) and 83.55 (3)° with respect to the thia­diazole and 4-methoxy­phenyl rings, respectively. In the crystal structure, inter­molecular C—H⋯O hydrogen bonds link the mol­ecules into centrosymmetric dimers.

## Related literature

For general background, see: Chen *et al.* (2000 ▶); Kidwai *et al.* (2000 ▶); Vicentini *et al.* (1998 ▶); Arun *et al.* (1999 ▶); Wasfy *et al.* (1996 ▶).
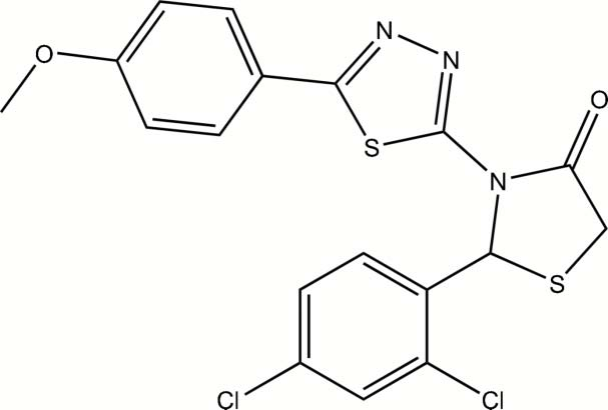


## Experimental

### 

#### Crystal data

C_18_H_13_Cl_2_N_3_O_2_S_2_
*M* *_r_* = 438.33
Triclinic, 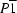

*a* = 7.1310 (14) Å
*b* = 8.1540 (16) Å
*c* = 16.671 (3) Å
α = 93.19 (3)°
β = 96.43 (3)°
γ = 105.89 (3)°
*V* = 922.7 (3) Å^3^
*Z* = 2
Mo *K*α radiation
μ = 0.60 mm^−1^
*T* = 298 (2) K
0.30 × 0.10 × 0.10 mm

#### Data collection

Enraf–Nonius CAD-4 diffractometer
Absorption correction: ψ scan (North *et al.*, 1968 ▶) *T*_min_ = 0.841, *T*_max_ = 0.943
3606 measured reflections
3315 independent reflections
2228 reflections with *I* > 2σ(*I*)
*R*_int_ = 0.084
3 standard reflections every 200 reflections intensity decay: none

#### Refinement

*R*[*F* ^2^ > 2σ(*F* ^2^)] = 0.067
*wR*(*F* ^2^) = 0.211
*S* = 1.02
3315 reflections
244 parameters
H-atom parameters constrained
Δρ_max_ = 0.47 e Å^−3^
Δρ_min_ = −0.61 e Å^−3^

### 

Data collection: *CAD-4 Software* (Enraf–Nonius, 1989 ▶); cell refinement: *CAD-4 Software*; data reduction: *XCAD4* (Harms & Wocadlo, 1995 ▶); program(s) used to solve structure: *SHELXS97* (Sheldrick, 2008 ▶); program(s) used to refine structure: *SHELXL97* (Sheldrick, 2008 ▶); molecular graphics: *SHELXTL* (Sheldrick, 2008 ▶); software used to prepare material for publication: *SHELXTL*.

## Supplementary Material

Crystal structure: contains datablocks global, I. DOI: 10.1107/S1600536808008465/hk2442sup1.cif

Structure factors: contains datablocks I. DOI: 10.1107/S1600536808008465/hk2442Isup2.hkl

Additional supplementary materials:  crystallographic information; 3D view; checkCIF report

## Figures and Tables

**Table 1:** Hydrogen-bond geometry (Å, °)

*D*—H⋯*A*	*D*—H	H⋯*A*	*D*⋯*A*	*D*—H⋯*A*
C4—H4*A*⋯S1	0.93	2.79	3.180 (7)	106
C6—H6*A*⋯N1	0.93	2.55	2.856 (8)	100
C12—H12*A*⋯Cl2	0.98	2.63	3.063 (5)	107
C14—H14*A*⋯N3	0.93	2.54	2.863 (8)	101
C14—H14*A*⋯O1^i^	0.93	2.41	3.219 (7)	146

Symmetry code: (i) 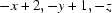.

**Fig. 1.**
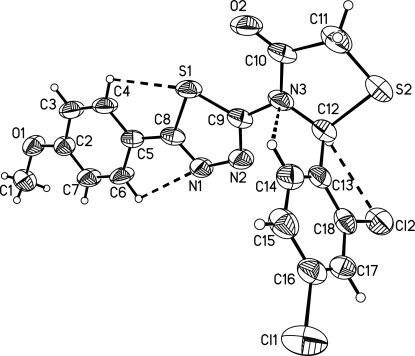
The molecular structure of the title molecule, with the atom-numbering scheme. Displacement ellipsoids are drawn at the 50% probability level. Hydrogen bonds are shown as dashed lines

**Fig. 2.**
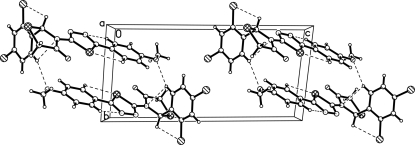
A partial packing diagram of (I). Hydrogen bonds are shown as dashed lines.

## supplementary crystallographic information

### Comment

1,3,4-Thiadiazole derivatives containing the thiazolidinone unit are of great interest because of their chemical and pharmaceutical properties. Some derivatives have fungicidal activities and exhibit certain herbicidal activities (Chen *et al.*,  2000; Kidwai *et al.*, 2000; Vicentini *et al.*,  1998). On the other hand, some of them show insecticidal activities (Arun *et al.*,  1999; Wasfy *et al.*,  1996). We report herein the crystal structure of the title compound, (I).

In the molecule of (I), (Fig. 1), rings A (C2-C7), B (S1/N1/N2/C8/C9) and D (C13-C18) are, of course, planar. The dihedral angles between them are A/B = 7.40 (4)°, A/D = 83.55 (3)° and B/D = 84.21 (4)°. So, rings A and B are nearly coplanar. Ring C (S2/N3/C10-C12) has envelope conformation with atom S2 displaced by 0.394 (3) Å from the plane of the other ring atoms. The intramolecular C-H···S, C-H···N and C-H···Cl hydrogen bonds (Table 1) result in the formation of two planar and two non-planar five-membered rings E (S1/C4/H4A/C5/C8), F (N1/C5/C6/H6A/C8) and G (N3/C12-C14/H14A), H (Cl2/C12/H12A/C13/C18). The dihedral angle between the planar rings E and F is E/F = 6.23 (3)°, and they are oriented with respect to the adjacent rings at dihedral angles of A/E = 3.26 (4)°, A/F = 4.55 (3)°, B/E = 5.03 (4)° and B/F = 7.27 (4)°. So, they are also nearly coplanar.

In the crystal structure, intermolecular C-H···O hydrogen bonds (Table 1) link the molecules into centrosymmetric dimers (Fig. 2), in which they may be effective in the stabilization of the structure.

### Experimental

*N*-(2,4-dichlorobenzylidene)-5-(4-methoxyphenyl)-1,3,4-thiadiazol -2-amine (5 mmol) and mercapto-acetic acid (5 mmol) were added in toluene (50 ml). The water was removed by distillation for 5 h. The reaction mixture was left to cool to room temperature, filtered, and the filter cake was crystallized from acetone to give pure compound (I) (m.p. 507-509 K). Crystals of (I) suitable for X-ray analysis were obtained by slow evaporation of an acetone solution.

### Refinement

H atoms were positioned geometrically, with C-H = 0.93, 0.98, 0.97 and 0.96 Å for aromatic, methine, methylene and methyl H, respectively, and constrained to ride on their parent atoms with U_iso_(H) = xU_eq_(C), where x = 1.5 for methyl H and x = 1.2 for all other H atoms.

### Crystal data

**Table:**

C_18_H_13_Cl_2_N_3_O_2_S_2_	*Z* = 2
*M**_r_* = 438.33	*F*_000_ = 448
Triclinic, *P*1	*D*_x_ = 1.578 Mg m^−^^3^
Hall symbol: -P 1	Melting point = 507–509 K
*a* = 7.1310 (14) Å	Mo *K*α radiation λ = 0.71073 Å
*b* = 8.1540 (16) Å	Cell parameters from 25 reflections
*c* = 16.671 (3) Å	θ = 9–12º
α = 93.19 (3)º	µ = 0.60 mm^−^^1^
β = 96.43 (3)º	*T* = 298 (2) K
γ = 105.89 (3)º	Block, colorless
*V* = 922.7 (3) Å^3^	0.30 × 0.10 × 0.10 mm

### Data collection

**Table:**

Enraf–Nonius CAD-4 diffractometer	*R*_int_ = 0.084
Radiation source: fine-focus sealed tube	θ_max_ = 25.2º
Monochromator: graphite	θ_min_ = 1.2º
*T* = 298(2) K	*h* = 0→8
ω/2θ scans	*k* = −9→9
Absorption correction: ψ scan(North *et al.*, 1968)	*l* = −19→19
*T*_min_ = 0.841, *T*_max_ = 0.943	3 standard reflections
3606 measured reflections	every 200 reflections
3315 independent reflections	intensity decay: none
2228 reflections with *I* > 2σ(*I*)	

### Refinement

**Table:**

Refinement on *F*^2^	Secondary atom site location: difference Fourier map
Least-squares matrix: full	Hydrogen site location: inferred from neighbouring sites
*R*[*F*^2^ > 2σ(*F*^2^)] = 0.067	H-atom parameters constrained
*wR*(*F*^2^) = 0.211	*w* = 1/[σ^2^(*F*_o_^2^) + (0.1*P*)^2^ + 2*P*] where *P* = (*F*_o_^2^ + 2*F*_c_^2^)/3
*S* = 1.02	(Δ/σ)_max_ < 0.001
3315 reflections	Δρ_max_ = 0.47 e Å^−^^3^
244 parameters	Δρ_min_ = −0.61 e Å^−^^3^
Primary atom site location: structure-invariant direct methods	Extinction correction: none

### Special details

**Table:**

Geometry. All e.s.d.'s (except the e.s.d. in the dihedral angle between two l.s. planes) are estimated using the full covariance matrix. The cell e.s.d.'s are taken into account individually in the estimation of e.s.d.'s in distances, angles and torsion angles; correlations between e.s.d.'s in cell parameters are only used when they are defined by crystal symmetry. An approximate (isotropic) treatment of cell e.s.d.'s is used for estimating e.s.d.'s involving l.s. planes.
Refinement. Refinement of F^2^ against ALL reflections. The weighted R-factor wR and goodness of fit S are based on F^2^, conventional R-factors R are based on F, with F set to zero for negative F^2^. The threshold expression of F^2^ > 2sigma(F^2^) is used only for calculating R-factors(gt) etc. and is not relevant to the choice of reflections for refinement. R-factors based on F^2^ are statistically about twice as large as those based on F, and R- factors based on ALL data will be even larger.

### Fractional atomic coordinates and isotropic or equivalent isotropic displacement parameters (Å^2^)

**Table:**

	*x*	*y*	*z*	*U*_iso_*/*U*_eq_	
Cl1	1.3375 (3)	0.6645 (2)	0.50289 (13)	0.0801 (6)	
Cl2	1.1228 (2)	1.20318 (19)	0.40205 (10)	0.0606 (5)	
S1	0.78625 (18)	0.7174 (2)	0.06058 (9)	0.0492 (4)	
S2	0.5812 (2)	0.9562 (2)	0.33166 (11)	0.0659 (5)	
O1	1.3277 (6)	0.6538 (6)	−0.2389 (2)	0.0616 (11)	
O2	0.4564 (5)	0.6490 (6)	0.1380 (3)	0.0653 (12)	
N1	1.1375 (6)	0.9039 (7)	0.1050 (3)	0.0535 (13)	
N2	1.0345 (6)	0.9262 (7)	0.1690 (3)	0.0557 (13)	
N3	0.7229 (6)	0.8433 (6)	0.2094 (3)	0.0488 (12)	
C1	1.5346 (9)	0.7003 (9)	−0.2423 (4)	0.0677 (18)	
H1B	1.5586	0.6672	−0.2955	0.102*	
H1C	1.5962	0.6431	−0.2030	0.102*	
H1D	1.5880	0.8220	−0.2306	0.102*	
C2	1.2658 (8)	0.6915 (7)	−0.1674 (3)	0.0481 (13)	
C3	1.0653 (8)	0.6308 (9)	−0.1639 (4)	0.0606 (17)	
H3A	0.9826	0.5675	−0.2088	0.073*	
C4	0.9884 (8)	0.6632 (8)	−0.0956 (4)	0.0556 (16)	
H4A	0.8538	0.6203	−0.0944	0.067*	
C5	1.1052 (7)	0.7578 (7)	−0.0282 (3)	0.0444 (13)	
C6	1.3061 (8)	0.8127 (8)	−0.0323 (4)	0.0561 (16)	
H6A	1.3900	0.8721	0.0131	0.067*	
C7	1.3842 (8)	0.7822 (8)	−0.1009 (4)	0.0556 (16)	
H7A	1.5189	0.8237	−0.1021	0.067*	
C8	1.0288 (7)	0.8015 (7)	0.0450 (3)	0.0451 (13)	
C9	0.8518 (7)	0.8390 (7)	0.1524 (3)	0.0457 (13)	
C10	0.5300 (7)	0.7452 (8)	0.1973 (4)	0.0491 (14)	
C11	0.4280 (9)	0.7736 (9)	0.2684 (4)	0.0670 (18)	
H11A	0.4038	0.6734	0.2987	0.080*	
H11B	0.3025	0.7928	0.2499	0.080*	
C12	0.7992 (7)	0.9486 (7)	0.2846 (3)	0.0463 (13)	
H12A	0.8693	1.0643	0.2731	0.056*	
C13	0.9363 (7)	0.8774 (7)	0.3396 (3)	0.0414 (12)	
C14	0.9118 (8)	0.7054 (7)	0.3390 (4)	0.0501 (14)	
H14A	0.8099	0.6322	0.3033	0.060*	
C15	1.0296 (8)	0.6354 (7)	0.3883 (4)	0.0518 (14)	
H15A	1.0095	0.5175	0.3865	0.062*	
C16	1.1798 (8)	0.7468 (8)	0.4411 (3)	0.0473 (13)	
C17	1.2064 (7)	0.9194 (7)	0.4448 (3)	0.0464 (13)	
H17A	1.3064	0.9927	0.4813	0.056*	
C18	1.0853 (7)	0.9832 (7)	0.3946 (3)	0.0425 (12)	

### Atomic displacement parameters (Å^2^)

**Table:**

	*U*^11^	*U*^22^	*U*^33^	*U*^12^	*U*^13^	*U*^23^
Cl1	0.0617 (10)	0.0839 (13)	0.0961 (14)	0.0270 (9)	−0.0084 (9)	0.0253 (10)
Cl2	0.0494 (8)	0.0463 (8)	0.0828 (11)	0.0091 (6)	0.0096 (7)	−0.0025 (7)
S1	0.0242 (6)	0.0653 (10)	0.0511 (9)	0.0018 (6)	0.0043 (6)	0.0040 (7)
S2	0.0417 (8)	0.0898 (13)	0.0728 (11)	0.0289 (8)	0.0138 (8)	−0.0028 (9)
O1	0.044 (2)	0.072 (3)	0.058 (3)	−0.001 (2)	0.0138 (19)	−0.011 (2)
O2	0.028 (2)	0.088 (3)	0.068 (3)	−0.001 (2)	0.0030 (19)	−0.002 (3)
N1	0.028 (2)	0.076 (3)	0.051 (3)	0.004 (2)	0.011 (2)	−0.005 (2)
N2	0.030 (2)	0.075 (3)	0.054 (3)	0.002 (2)	0.008 (2)	−0.003 (3)
N3	0.026 (2)	0.072 (3)	0.049 (3)	0.012 (2)	0.012 (2)	0.007 (2)
C1	0.045 (3)	0.090 (5)	0.071 (4)	0.018 (3)	0.026 (3)	0.002 (4)
C2	0.038 (3)	0.049 (3)	0.053 (3)	0.004 (2)	0.009 (3)	0.006 (3)
C3	0.033 (3)	0.084 (5)	0.053 (4)	0.004 (3)	0.000 (3)	−0.010 (3)
C4	0.025 (3)	0.076 (4)	0.057 (4)	0.003 (3)	−0.001 (2)	−0.002 (3)
C5	0.029 (3)	0.048 (3)	0.052 (3)	0.003 (2)	0.004 (2)	0.005 (3)
C6	0.027 (3)	0.076 (4)	0.050 (3)	−0.005 (3)	0.001 (2)	−0.009 (3)
C7	0.029 (3)	0.075 (4)	0.055 (4)	0.001 (3)	0.009 (3)	0.001 (3)
C8	0.025 (2)	0.052 (3)	0.056 (3)	0.004 (2)	0.007 (2)	0.011 (3)
C9	0.030 (3)	0.056 (3)	0.052 (3)	0.012 (2)	0.007 (2)	0.011 (3)
C10	0.025 (3)	0.061 (4)	0.062 (4)	0.010 (2)	0.008 (3)	0.012 (3)
C11	0.039 (3)	0.092 (5)	0.072 (4)	0.018 (3)	0.020 (3)	0.008 (4)
C12	0.034 (3)	0.054 (3)	0.054 (3)	0.015 (2)	0.010 (2)	0.006 (3)
C13	0.028 (2)	0.048 (3)	0.049 (3)	0.011 (2)	0.011 (2)	0.000 (2)
C14	0.037 (3)	0.052 (3)	0.058 (4)	0.009 (3)	0.005 (3)	−0.003 (3)
C15	0.041 (3)	0.043 (3)	0.071 (4)	0.011 (3)	0.014 (3)	0.004 (3)
C16	0.035 (3)	0.058 (4)	0.053 (3)	0.016 (3)	0.010 (2)	0.011 (3)
C17	0.028 (3)	0.057 (4)	0.050 (3)	0.006 (2)	0.006 (2)	0.000 (3)
C18	0.034 (3)	0.042 (3)	0.054 (3)	0.010 (2)	0.019 (2)	0.003 (2)

### Geometric parameters (Å, °)

**Table:**

S1—C9	1.721 (6)	C4—C5	1.375 (8)
S1—C8	1.732 (5)	C4—H4A	0.9300
Cl1—C16	1.734 (6)	C5—C6	1.390 (7)
O1—C2	1.366 (7)	C5—C8	1.456 (8)
O1—C1	1.427 (7)	C6—C7	1.366 (8)
N1—C8	1.295 (7)	C6—H6A	0.9300
N1—N2	1.393 (6)	C7—H7A	0.9300
C1—H1B	0.9600	C10—C11	1.498 (8)
C1—H1C	0.9600	C11—H11A	0.9700
C1—H1D	0.9600	C11—H11B	0.9700
Cl2—C18	1.735 (5)	C12—C13	1.517 (7)
S2—C11	1.788 (7)	C12—H12A	0.9800
S2—C12	1.832 (5)	C13—C14	1.364 (8)
O2—C10	1.200 (7)	C13—C18	1.381 (7)
C2—C7	1.358 (8)	C14—C15	1.367 (8)
C2—C3	1.388 (7)	C14—H14A	0.9300
N2—C9	1.292 (7)	C15—C16	1.383 (8)
N3—C10	1.375 (7)	C15—H15A	0.9300
N3—C9	1.399 (7)	C16—C17	1.365 (8)
N3—C12	1.441 (7)	C17—C18	1.360 (8)
C3—C4	1.362 (8)	C17—H17A	0.9300
C3—H3A	0.9300		
			
C9—S1—C8	86.0 (3)	N2—C9—S1	116.2 (4)
C2—O1—C1	117.6 (5)	N3—C9—S1	124.3 (4)
C8—N1—N2	113.1 (4)	O2—C10—N3	124.0 (5)
O1—C1—H1B	109.5	O2—C10—C11	125.4 (5)
O1—C1—H1C	109.5	N3—C10—C11	110.6 (5)
H1B—C1—H1C	109.5	C10—C11—S2	108.7 (4)
O1—C1—H1D	109.5	C10—C11—H11A	110.0
H1B—C1—H1D	109.5	S2—C11—H11A	110.0
H1C—C1—H1D	109.5	C10—C11—H11B	110.0
C11—S2—C12	92.5 (3)	S2—C11—H11B	110.0
C7—C2—O1	125.2 (5)	H11A—C11—H11B	108.3
C7—C2—C3	118.7 (5)	N3—C12—C13	112.7 (4)
O1—C2—C3	116.1 (5)	N3—C12—S2	104.8 (3)
C9—N2—N1	110.6 (5)	C13—C12—S2	111.2 (4)
C10—N3—C9	122.2 (5)	N3—C12—H12A	109.3
C10—N3—C12	119.5 (4)	C13—C12—H12A	109.3
C9—N3—C12	118.3 (4)	S2—C12—H12A	109.3
C4—C3—C2	120.7 (5)	C14—C13—C18	117.3 (5)
C4—C3—H3A	119.7	C14—C13—C12	121.2 (5)
C2—C3—H3A	119.7	C18—C13—C12	121.5 (5)
C3—C4—C5	121.5 (5)	C13—C14—C15	123.3 (5)
C3—C4—H4A	119.2	C13—C14—H14A	118.4
C5—C4—H4A	119.2	C15—C14—H14A	118.4
C4—C5—C6	116.7 (5)	C14—C15—C16	117.3 (5)
C4—C5—C8	123.5 (5)	C14—C15—H15A	121.4
C6—C5—C8	119.8 (5)	C16—C15—H15A	121.4
C7—C6—C5	122.1 (5)	C17—C16—C15	121.2 (5)
C7—C6—H6A	119.0	C17—C16—Cl1	119.7 (4)
C5—C6—H6A	119.0	C15—C16—Cl1	119.0 (5)
C2—C7—C6	120.2 (5)	C18—C17—C16	119.4 (5)
C2—C7—H7A	119.9	C18—C17—H17A	120.3
C6—C7—H7A	119.9	C16—C17—H17A	120.3
N1—C8—C5	122.9 (4)	C17—C18—C13	121.5 (5)
N1—C8—S1	114.1 (4)	C17—C18—Cl2	117.8 (4)
C5—C8—S1	123.0 (4)	C13—C18—Cl2	120.6 (4)
N2—C9—N3	119.5 (5)		
			
C1—O1—C2—C7	5.7 (9)	C12—N3—C10—O2	−177.0 (6)
C1—O1—C2—C3	−174.3 (6)	C9—N3—C10—C11	179.2 (5)
C8—N1—N2—C9	−0.6 (7)	C12—N3—C10—C11	2.1 (7)
C7—C2—C3—C4	0.7 (10)	O2—C10—C11—S2	−169.0 (5)
O1—C2—C3—C4	−179.3 (6)	N3—C10—C11—S2	12.0 (7)
C2—C3—C4—C5	0.6 (10)	C12—S2—C11—C10	−17.2 (5)
C3—C4—C5—C6	−2.5 (9)	C10—N3—C12—C13	106.6 (5)
C3—C4—C5—C8	177.4 (6)	C9—N3—C12—C13	−70.6 (6)
C4—C5—C6—C7	3.2 (10)	C10—N3—C12—S2	−14.5 (6)
C8—C5—C6—C7	−176.8 (6)	C9—N3—C12—S2	168.3 (4)
O1—C2—C7—C6	179.9 (6)	C11—S2—C12—N3	17.6 (4)
C3—C2—C7—C6	−0.1 (10)	C11—S2—C12—C13	−104.5 (4)
C5—C6—C7—C2	−1.9 (10)	N3—C12—C13—C14	−31.0 (7)
N2—N1—C8—C5	−179.4 (5)	S2—C12—C13—C14	86.3 (6)
N2—N1—C8—S1	−0.9 (7)	N3—C12—C13—C18	151.9 (5)
C4—C5—C8—N1	−173.3 (6)	S2—C12—C13—C18	−90.8 (5)
C6—C5—C8—N1	6.6 (9)	C18—C13—C14—C15	−1.7 (8)
C4—C5—C8—S1	8.3 (8)	C12—C13—C14—C15	−178.9 (5)
C6—C5—C8—S1	−171.8 (5)	C13—C14—C15—C16	0.1 (9)
C9—S1—C8—N1	1.5 (5)	C14—C15—C16—C17	1.4 (8)
C9—S1—C8—C5	−179.9 (5)	C14—C15—C16—Cl1	−178.2 (4)
N1—N2—C9—N3	179.6 (5)	C15—C16—C17—C18	−1.3 (8)
N1—N2—C9—S1	1.8 (7)	Cl1—C16—C17—C18	178.3 (4)
C10—N3—C9—N2	−175.9 (5)	C16—C17—C18—C13	−0.3 (8)
C12—N3—C9—N2	1.1 (8)	C16—C17—C18—Cl2	179.4 (4)
C10—N3—C9—S1	1.7 (8)	C14—C13—C18—C17	1.7 (8)
C12—N3—C9—S1	178.7 (4)	C12—C13—C18—C17	179.0 (5)
C8—S1—C9—N2	−1.9 (5)	C14—C13—C18—Cl2	−178.0 (4)
C8—S1—C9—N3	−179.6 (5)	C12—C13—C18—Cl2	−0.7 (7)
C9—N3—C10—O2	0.1 (9)		

### Hydrogen-bond geometry (Å, °)

**Table:**

*D*—H···*A*	*D*—H	H···*A*	*D*···*A*	*D*—H···*A*
C4—H4A···S1	0.93	2.79	3.180 (7)	106
C6—H6A···N1	0.93	2.55	2.856 (8)	100
C12—H12A···Cl2	0.98	2.63	3.063 (5)	107
C14—H14A···N3	0.93	2.54	2.863 (8)	101
C14—H14A···O1^i^	0.93	2.41	3.219 (7)	146

Symmetry codes: (i) −*x*+2, −*y*+1, −*z*.
